# Miscellany

**Published:** 1911-07

**Authors:** 


### MISCELLANY

### The Suprarenal Situation

When Vulpian, a French chemist, in 1856, reported that the suprarenal glands of mammals contained a peculiar substance giving certain color reactions with ferric chloride, iodine, and alkalies, and quickly changing in contact with the air and on exposure to light, little might anyone have expected that fifty-five years later this peculiar substance would be the subject of a product patent.

In 1904 the H. K. Mulford Company placed upon the market adrin, its brand of epinephrine, the active principle of the adrenal gland, believing that the pioneer work done by von Fürth and Abel justified it in doing so,—and that a product patent on the active principle existing in nature could not possibly be upheld, particularly in view of the fact that its existence has been recognised for fifty years; that nearly all of its chemical reactions and properties were previously known and described; that its chemical nature had been accurately predicted; that its medicinal virtues had been discovered and put into practical use; and that it had been actually isolated in various degrees of purity in the form of a benzoylated derivative and in the form of a zinc and an iron compound.

Moreover; the H. K. Mulford Company, recognising that the first object of the patent law is to "promote progress in the sciences and arts" believed and still believes that the granting of "product patents" on medicinal substances, whether or not they exist pre-formed in nature, are a hindrance to, rather than a means of promoting progress in the practice of medicine, and used their efforts to defeat a product patent which it deemed to be not only contrary to the object and spirit of the patent law but contrary to the best interests of pharmacologic practice in the United States.

On April 29, 1911, Judge Hand, in the United States Circuit Court for the southern district of New York, handed down a decision sustaining certain of the patent claims of Dr. Takamine and declaring H. K. Mulford Company products to infringe these claims.

The H. K. Mulford Company wishes to call attention to the fact that in defending these suits it has consistently and at great cost endeavored to uphold its antagonistic position toward the product patent for medicinal substances, believing that product patents on all substances used in medicine, work an injustice on the medical and pharmaceutical professions and are inimical to the public good.

The court having decided that the manufacture of Adrin, the Mulford brand of epinephrine (the active principle of the adrenal glands,) conflicts with the product patents granted to Dr. Takamine, the H. K. Mulford Company will discontinue its manufacture in the form of solution, tablets and hypodermics, until their appeal is decided in the higher court. Other preparations which have contained the adrian brand of epinephrine will be prepared with an amount of purified extract of adrenals equivalent to the active principle contained in the glands.

---

The New York State Civil Service Commission will hold, on or about August 5, 1911, an examination for Superintendent of Syracuse State Institution for Feeble Minded Children. Open to men only. Salary $4,000 a year, with maintenance for superintendent and his family. Candidates must be well trained physicians, and should also have had experience in teaching and in administrative work. Application blanks may be obtained by addressing State Civil Service Commission, Albany, N. Y., and all applications must be filed with the State Civil Service Commission not later than July 28, 1911.

---

Dr. Howard W. Longyear, of Detroit, a member of the American Association of Obstetricians and Gynecologists, has invented an abdominal supporter especially designed for holding floating kidneys in their normal position. It is manufactured by the J. F. Hartz Co., of Detroit, and is well worth the consideration of the medical profession.

---

Battle & Co., of St. Louis, have just issued No. 16 of their series of charts on dislocations. This series forms a most valuable and interesting addition to any physician's library. They will be sent free of charge on application, and back numbers will also be supplied.

---

### Additional News Items

At a meeting of the Board of Regents of the University of the State of New York, held at Albany, June 22, Dr. P. Hanson Hiss, Jr., of Columbia College, was appointed examiner in bacteriology, state board of medical examiners.

Dr. Glentworth R. Butler, of Brooklyn, examiner in diagnosis, has been elected president of the New York state board of medical examiners.

